# Restoration of the hip geometry after two-stage exchange with intermediate resection arthroplasty for periprosthetic joint infection

**DOI:** 10.1038/s41598-021-84692-x

**Published:** 2021-03-04

**Authors:** Jan Hubert, Frank Timo Beil, Tim Rolvien, Christian Ries, Stephan Frosch, Dominik Saul, Thelonius Hawellek

**Affiliations:** 1 Department of Trauma Surgery, Orthopaedics and Plastic Surgery, University Medical Center Göttingen, Göttingen, Germany; 2 Department of Orthopaedics, University Medical Center Hamburg-Eppendorf, Martinistraße 52, 20246 Hamburg, Germany; 3 Kogod Center On Aging and Division of Endocrinology, Mayo Clinic, Rochester, MN 55905 USA

**Keywords:** Outcomes research, Bone, Bone

## Abstract

Two-stage exchange with intermediate resection arthroplasty (RA) is a well-established surgical procedure in the treatment of chronic periprosthetic joint infection (PJI), whereby a higher failure rate of final hip geometry restoration due to tissue contraction is controversially discussed. The aim was to evaluate radiographic changes of hip geometry parameters during PJI treatment and to determine the impact of the intermediate RA on the final joint restoration after reimplantation of a total hip arthroplasty (reTHA). Radiographic parameters (leg length (LL), femoral offset (FO), horizontal/vertical acetabular center of rotation distance (h/vCORD)) of 47 patients (mean age: 64.1 years) were measured on standard radiographs of the pelvis and compared between four different stages during PJI treatment (pre-replacement status (preTHA), primary total hip arthroplasty (pTHA), RA and reTHA). The RA duration (mean: 10.9 months) and the number of reoperations during this period (mean: n = 2.0) as well as their impact on hip geometry restoration were evaluated. Between preTHA and pTHA/reTHA an equivalent restoration was measured regarding the FO (*p* < 0.001/*p* < 0.001) and hCORD (*p* = 0.016/*p* < 0.001), but not regarding the LL and vCORD. In contrast, analysis revealed no influence of RA and an equivalent reconstruction of LL (*p* = 0.003), FO (*p* < 0.001), v/hCORD (*p* = 0.039/*p* = 0.035) at reTHA compared to pTHA. Furthermore, RA duration (*p* = 0.053) and the number of reoperations after RA (*p* = 0.134) had no impact on radiographic hip geometry restoration. The two-stage exchange with intermediate RA does not alter the preexisting hip joint parameters, whereby a good restoration of the final hip geometry, independent of the duration or the number of reoperations, can be achieved.

## Introduction

Periprosthetic joint infection (PJI) is a severe complication after primary total hip arthroplasty (pTHA) and occurs up to 1% of the cases^1–4^. PJI treatment can necessitate multiple operations with a long period of disability^5,6^, resulting in an enormous socioeconomic burden^2^. Although failure rates are high, the current gold standard for PJI treatment is a two-stage revision^7,8^. There is a consensus that one-stage revision is valuable, if a single, non-multidrug-resistant infecting microorganism is preoperatively identified and effective antibiotics are available^7^. In contrast, relative contraindications are the presence of a sinus tract or severe soft-tissue involvement^7^. However, in most cases a two-stage exchange is favored^9–11^, with success rates of eradication of over 80%^12–15^. The first part of this procedure includes the removal of all prosthetic components including cement residue, surgical sutures and other artificial materials. Subsequently followed by an extensive debridement, an intermediate resection arthroplasty (RA)^16^ or the placement of a temporary, antibiotic-loaded cement-spacer can be performed in the prosthesis-free interval before reimplantation. Although an equal infection control is described for both methods^17,18^, the use of an intermediate cement-spacer can improve the functional outcome by preserving the leg length as well as patients’ mobility in the interim period^17,19^, whereby a tissue contraction could be prevented^9,14,19^. Typical disadvantages are the occurrence of acetabular defects (which require a complex reconstruction or special implants), dislocation and migration of the spacer to the pelvis, but also fractures of the spacer as well as femoral fractures during spacer removal^18,20–22^. In addition, recent studies showed a persistent infection due to the adherence of microorganisms on the spacer as microbiological colonization could be confirmed by sonication in up to 50% after explantation^23,24^.

Complications reported to be associated with RA are (1) a difficult identification of intraoperative landmarks during reimplantation of a THA (reTHA) due to cicatricial retraction and muscular contractures^25^, (2) a disuse osteoporosis, which can impair the mechanical conditions for sufficient prosthesis fixation or can predispose to fractures^26^ and (3) a change of postoperative hip geometry, as leg length discrepancy (LLD) was frequently reported^25,27^.

To provide the best functional outcome for the patient, the main goal of primary and revision arthroplasty is to restore the anatomical and biomechanical conditions of the hip joint. Considering that these conditions are fully dissolved after RA, a reimplantation can be technically challenging concerning the restoration of hip joint geometry, especially the acetabular center of rotation (COR), leg length (LL) and femoral offset (FO)^28,29^. The restoration of hip geometry is influenced by the intraoperative periarticular tissue release but also by the accuracy of component positioning, which has an essential impact on muscle^30–35^ and hip function^36–40^, stability of the joint^34,35^ as well as the durability of the implanted components^30,41–46^. Therefore, special attention has to be given to a perfect positioning of the prosthesis components to the individual predefined anatomical and biomechanical conditions.

To our knowledge, only a few studies analyzed the restoration of radiographic parameters of hip joint geometry in patients who underwent a two-stage procedure with intermediate RA. Although they reported that the enormous preexisting LLD during RA could be significantly reduced after reTHA^27,29^, until today, further parameters like COR or FO, which can also influence the hip geometry and function, have not been analyzed. Moreover, there is no information regarding the reconstruction of hip geometry in dependence of RA duration, especially for LL and FO. It is assumed that RA leads to tissue contraction, however, studies that prove this hypothesis are missing and there is no information if RA duration can influence an adequate hip geometry restoration. Further information lacking in the literature is, if the number of reoperations during RA, which are frequently required, can promote tissue contraction and have a significant impact on hip joint restoration.

The final unknown fact is, if the initial pre-replacement “healthy” hip geometry (preTHA) has already been changed after primary THA. In the literature, only the final hip geometry was analyzed after reTHA, but it is conceivable that the pre-replacement LL, FO and COR could already be changed after primary THA. In order to make an accurate statement about the influence of RA on the final parameters after reTHA, the knowledge about the radiographic changes after primary THA is essential.

Therefore, the goal of this study was to analyze the quality of hip joint restoration based on radiographic parameters (COR, LL and FO) by standardized X-Rays in patients who underwent a two-stage exchange with intermediate RA in comparison to the initial pre-replacement hip joint and the hip geometry after pTHA. Furthermore, we evaluated if the duration of the applied RA or the number of reoperations before final reTHA have an impact on the radiographic hip joint restoration.

## Patients and methods

This retrospective study was approved by the local Ethics Committee (approval no. 22/12/17) and was carried out according to existing rules and regulations of the University Medical Center Göttingen.

### Study cohort

47 patients (n = 23 female and n = 24 male, mean age at time of RA: 64.1 years, SD ± 12.9, range 41–88) with primary chronic periprosthetic hip joint infection, who underwent a surgical two-stage exchange with intermediate RA between 2000 and 2016 were included in this retrospective study. The diagnosis of PJI included a combination of clinical findings, serologic markers, synovial fluid analysis, microbiological analysis and radiographic diagnostics. The surgery was performed by different, but experienced senior orthopedic surgeons in a single institution. The acetabular and femoral bone defects according to the Paprosky classification^47,48^ were classified prior to reimplantation (Table 1) and the chosen implants were specifically selected to the individual anatomical and biomechanical conditions of the patient, as special attention was given to a perfect positioning of the components.

**Table 1:** The biometric data of the study population.

Patient	Sex	Age at RA [y]	Duration (days) between		Number (n) of reoperations		Bone defects	
			pTHA and RA	RA and reTHA	After RA	After reTHA	Acetabular	Femoral
1	M	63	1271	363	2	2	3	–
2	M	78	381	287	1	–	1	–
3	M	46	1182	82	1	3	2	1
4	M	68	629	163	1	–	1	–
5	M	41	4174	123	–	–	2	–
6	F	65	5103	2193	7	–	1	–
7	F	46	5215	119	–	–	2	2
8	F	52	5906	308	5	1	2	2
9	F	65	3832	195	1	–	1	–
10	M	76	2220	125	3	1	–	2
11	M	45	406	761	–	–	–	–
12	F	65	3278	509	3	1	1	–
13	F	76	377	90	–	–	1	–
14	F	57	978	56	–	–	1	–
15	F	75	1875	70	1	–	1	–
16	M	44	2735	293	1	2	2	–
17	M	65	689	219	1	–	1	1
18	F	77	464	106	2	1	2	1
19	F	81	6629	118	–	–	1	1
20	M	72	827	464	8	1	1	–
21	M	75	328	392	3	–	–	–
22	F	56	3437	375	3	–	1	2
23	F	67	1427	415	1	–	3	2
24	F	61	3893	79	–	–	3	1
25	F	66	786	889	6	1	1	–
26	F	56	551	588	4	–	1	–
27	F	51	5430	101	–	–	3	2
28	M	74	1502	440	2	–	1	–
29	M	65	597	302	2	1	2	–
30	M	64	797	55	1	–	–	–
31	M	55	245	270	3	–	2	–
32	M	71	338	112	–	1	1	–
33	F	56	1037	315	2	–	–	–
34	F	56	8939	140	2	–	1	–
35	F	88	3374	272	2	1	–	–
36	M	80	4899	494	5	2	2	–
37	F	82	3183	398	6	1	–	3
38	F	57	596	87	2	–	–	–
39	M	86	534	72	1	–	1	–
40	M	47	820	67	–	1	1	–
41	F	48	912	88	1	–	1	–
42	M	49	939	172	2	1	–	–
43	F	59	877	784	3	2	2	–
44	M	80	2952	823	5	1	1	2
45	M	47	634	254	1	–	1	–
46	M	74	1819	180	–	1	–	2
47	M	84	1790	740	2	1	–	–

***M ***male ***F*** female ***RA ***resection arthroplasty ***pTHA*** primary total hip arthroplasty ***reTHA*** reimplantation of THA.

In addition, the implant type was adapted to the extent of acetabular and/or femoral bone defects. The acetabular defects were treated in type I: with standard acetabular components, in type II: with standard/jumbo acetabular components or reinforcement cages and in type III: with reinforcement cages. The femoral defects were treated in type I–II: with standard or long femoral monoblock stems and in type III–IV: with long monoblock or modular revision stems.

Reimplantation was only performed when (1) the wound and infection had completely healed and (2) the patient's general condition was suitable. In some cases, due to the patient's poor condition, surgery could only be performed several months later.

Patients with bilateral THA were excluded from this study. Only patients with standardized radiographic images of the pelvis at four different stages during PJI treatment (1. pre-replacement (preTHA), 2. after primary total hip arthroplasty (pTHA), 3. after RA and 4. after reimplantation of a THA (reTHA)) were included in this study.

The RA duration (mean: 10.9 months, SD ± 11.9, range: 1.8–73.1) and the number of reoperations (mean: n = 2.0, SD ± 2.0, range: 0–8) during this period were registered. Reoperations during the RA period were performed in cases of persistent infection or wound healing disorders. The biometric data of the patients are listed in Table 1.

### Radiographic measurements

**Figure 1:**
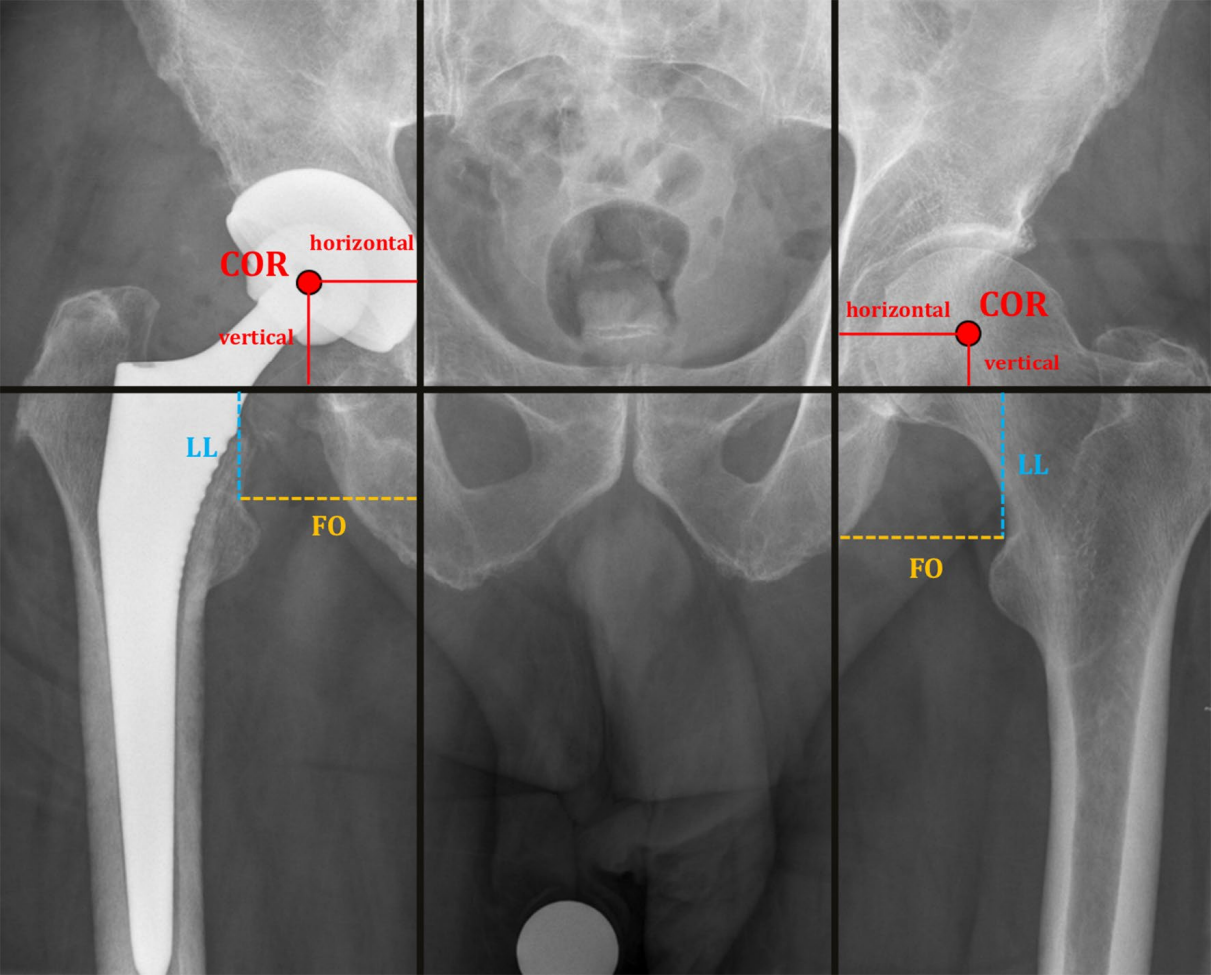
Standardized radiographs of the pelvis demonstrating the technique for measurement of the different radiographic parameters (leg length (LL), femoral offset (FO), horizontal and vertical acetabular center of rotation distance (hCORD, vCORD)) during PJI treatment [in mm]. **LL** (blue line) was measured as the length between the interteardrop line and the lesser trochanter. **FO** (yellow line) was measured as the length between the teardrop and the lesser trochanter. The **vCORD** and **hCORD** (red lines) were measured as the length between the teardrop and the center of rotation (COR).

Standardized radiographs of the pelvis with a reference ball of 25 mm in anteroposterior direction were analyzed. Each radiographic image was centered on the pubic symphysis (equal size of the obturator foramens) and included both proximal femora. For each patient four different radiographic parameters (leg length (LL), femoral offset (FO), horizontal and vertical acetabular center of rotation distance (hCORD, vCORD)) were measured [in mm] in the same manner as previously published^49^ at four different stages during PJI treatment (Fig. 1). The radiographic parameters after pTHA and reTHA were evaluated on the first postoperative standardized radiographs. The initial pre-replacement status of the hip was recorded before pTHA and the RA parameters of the hip were determined on the radiographs immediately before reTHA. The measurements were performed on high-resolution monitors with diagnostic PACS (Picture Archiving and Communication System, Centricity PACS, General Electric Company Healthcare Systems) by two independent observers who were blinded to the other results.

#### Statistical analysis

The biometric data of the patients are reported as mean values ± standard deviations (SD). Group comparisons were conducted by Wilcoxon rank-sum or Kruskal–Wallis test. *P*-values less than 0.05 were considered statistically significant. In our analysis, a significant *p*-value means equivalence between the measurements and a sufficient restoration of the radiographic parameters, whereas non-significant *p*-values mean the two measurements are not equivalent (insufficient restoration of the radiographic parameters). Pearson's r and Kendall's tau correlation coefficients were calculated. Backward variable selection was applied to multiple linear regression models in order to assess the effect on the difference between preTHA and pTHA/reTHA as well as between pTHA and reTHA. Furthermore, the impact of the duration between RA and reTHA and the number of reoperations during this period on the radiographic parameters of reTHA was evaluated in this model. The significance level was set to alpha = 5% for all statistical tests. A *p*-value less than 0.05 was considered statistically significant. In case of multiple testing, raw *p*-values were adjusted by the Bonferroni-Holm method. All analyses were performed with the statistic software R (version 3.4.0)^50^.

## Results

### Change of the radiographic parameters of hip geometry during PJI treatment

**Figure 2:**
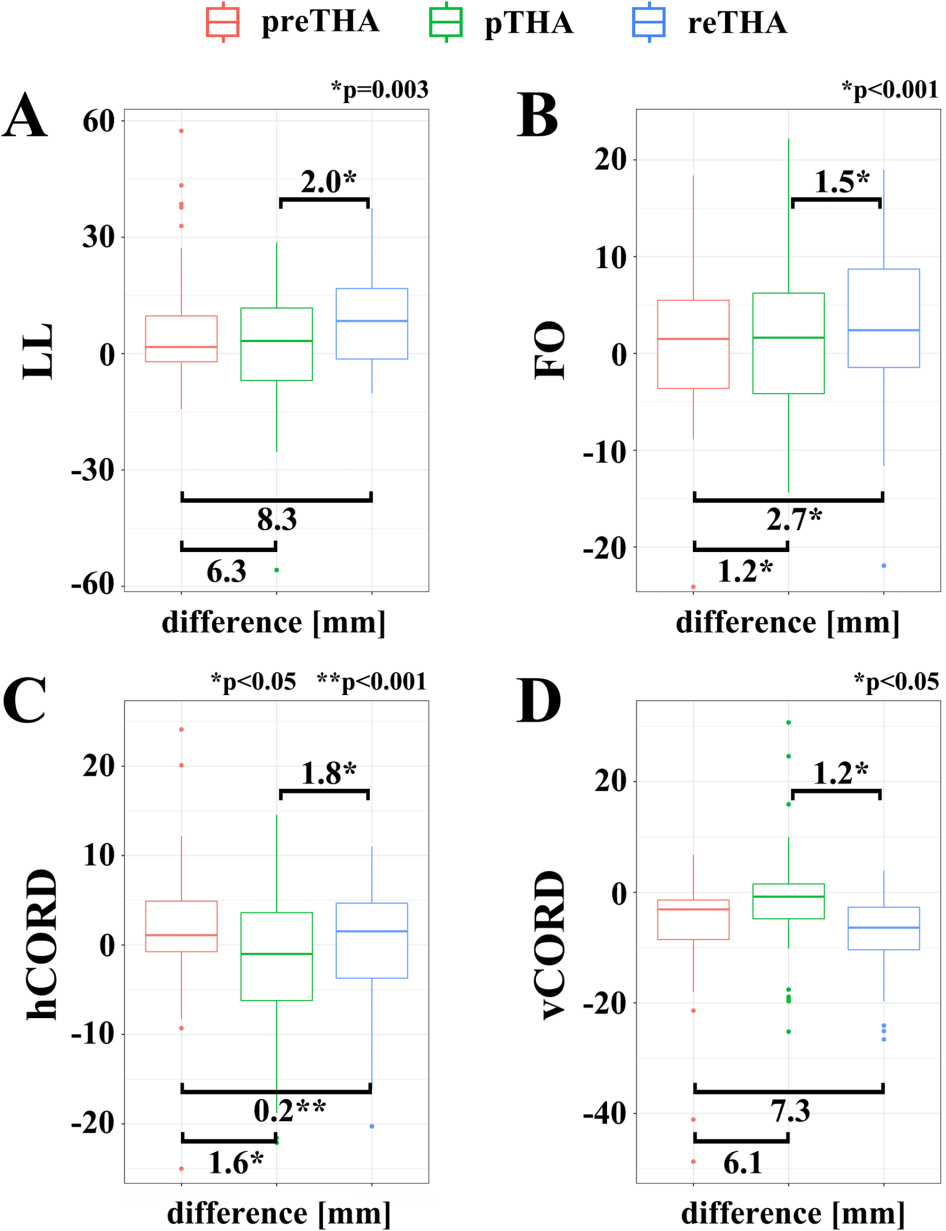
Box plots showing changes of the radiographic parameters (***LL*** leg length ***FO ***femoral offset ***h/vCORD*** horizontal/vertical center of rotation distance) [mean difference in mm] of the hip geometry at different stages during PJI treatment (***preTHA*** pre-replacement status ***pTHA*** primary total hip arthroplasty ***reTHA*** reimplantation of THA).

The changes of the evaluated radiographic parameters at different stages during PJI treatment are shown in Table 2 and in Fig. 2A–D.

**Table 2:** Radiographic parameters (mean ± SD) [in mm] of hip geometry at different stages during PJI treatment**.**

	preTHA		pTHA		RA		reTHA	
	Mean	SD	Mean	SD	Mean	SD	Mean	SD
LL	46.4	± 8.7	40.2	± 14.8	− 7.7	± 15.3	38.1	± 13.2
FO	49.9	± 6.5	48.7	± 8.4	52.9	± 8.0	47.2	± 6.7
hCORD	37.8	± 5.7	36.2	± 8.5			38.0	± 7.6
vCORD	16.4	± 3.3	22.5	± 10.7			23.7	± 7.5

***LL ***leg length ***FO ***femoral offset ***h/vCORD*** horizontal/vertical center of rotation distance ***preTHA*** pre-replacement status ***pTHA*** primary total hip arthroplasty ***RA ***resection arthroplasty ***reTHA*** reimplantation of THA.

#### Leg length (LL) [in mm]

The final LL after reTHA was not equivalent (*p* = 0.51) in comparison to the initial preTHA LL with a mean reduction of 8.3 mm, 95%-CI [5.3, 11.3]) (Fig. 2A). However, a LL difference was already measured after pTHA with a mean LL reduction of 6.3 mm, 95%-CI [2.6, 9.9]) and was also not equal to the initial preTHA LL (*p* = 0.19).

In contrast, comparing the radiographic LL between pTHA and reTHA, statistical analysis revealed no influence of the applied RA and an equivalent restoration (*p* = 0.003) with a marginal mean reduction of 2.0 mm (95%-CI [− 1.6, 5.6]) after reTHA.

#### Femoral offset (FO) [in mm]

The final FO after reTHA was equivalent (*p* < 0.001) to the initial preTHA with a mean reduction of 2.7 mm, 95%-CI [0.8, 4.7] (Fig. 2B). An equivalent FO restoration (*p* < 0.001) was also detected between preTHA and pTHA (mean reduction: 1.2 mm, 95%-CI [− 0.7, 3.1]).

Comparing the radiographic FO between pTHA and reTHA, an equivalent restoration was achieved after RA (*p* < 0.001), with a marginal mean reduction of 1.5 mm, 95%-CI [− 0.5, 3.5]. Therefore, the applied RA did not alter the restoration of the FO.

#### Horizontal center of rotation distance (hCORD) [in mm]

The mean hCORD after RA was not measured at this stage. In comparison to preTHA, statistical analysis revealed an equivalent hCORD restoration after pTHA (mean decrease of 1.6 mm, 95%-CI [− 0.3; 3.5], *p* = 0.016) but also after reTHA (mean increase: − 0.2 mm, 95%-CI [− 2.0, 1.6], *p* < 0.001) (Fig. 2C). Here again, the applied intermediate RA did not alter the restoration of the hCORD, whereby nearly equivalent values were registered between pTHA and reTHA (*p* = 0.035) with a mean increase of − 1.8 mm (95%-CI [− 3.7, 0.2]).

#### Vertical center of rotation distance (vCORD) [in mm]

The mean vCORD after RA was not evaluated at this stage. A change of the vCORD (mean increase: − 7.3 mm, 95%-CI [− 9.0, − 5.6]) was observed after reTHA and was not equivalent to the preTHA (*p* = 1) (Fig. 2D). This observation was already registered after pTHA (mean increase: − 6.1 mm, 95%-CI [− 8.5, − 3.6]) and was also not equivalent in comparison to the initial preTHA (*p* = 1). In contrast, comparing the radiographic status between pTHA and reTHA, statistical analysis revealed no influence of the applied RA and an equivalent restoration of vCORD (*p* = 0.04) with a marginal increase of mean 1.2 mm, 95%-CI [− 3.6, 1.2]).

### RA duration and reoperations: impact on the restoration of LL and FO

Based on the fact that tissue contraction is generally a time dependent process, the duration of the applied RA until reTHA was registered. Furthermore, the number of reoperations, which can also influence tissue contraction due to formation of scar tissue was evaluated. The impact on the restoration of LL and FO (as dependent variables) was evaluated with a multiple linear regression model. The mean RA duration was 10.9 months (SD ± 11.8, range 1.8–72.1) and the mean number of reoperations during RA period was n = 2.0 (SD ± 2.0, range 0–8). The multiple linear regression model revealed no impact of RA duration or the number of reoperations on the restoration of LL (*p* = 0.97/*p* = 0.35) or FO (*p* = 0.32/*p* = 0.75) (Table 3).

**Table 3:** Multiple linear regression model. Dependent variables (LL and FO) and independent variables (RA duration and number of reoperations before reTHA).

Independent variables	LL		FO	
	Estimate	*p*-value	Estimate	*p*-value
RA duration	− 0.0003	0.97	− 0.004	0.32
Number of reoperations	1.467	0.35	− 0.2466	0.75

***LL ***leg length **FO** femoral offset ***RA ***resection arthroplasty ***reTHA*** reimplantation of THA.

## Discussion

In this retrospective study, the quality of hip joint geometry restoration based on radiographic parameters was analyzed after two-stage exchange with intermediate RA at different stages during PJI treatment. The RA duration and the number of reoperations during this period as well as their impact on the final hip geometry restoration were evaluated.

The main goal of primary and revision arthroplasty is to achieve a perfect restoration of the anatomical and biomechanical conditions of the hip joint to ensure an adequate muscle and hip function^30–40^, a sufficient joint stability^34,35^ and to provide the best functional outcome, which can positively influence the durability of the implants^30,41–46^. The restoration of the hip joint geometry after RA is surgically challenging and the reimplantation can be technically sophisticated^25–27^. Recent studies reported that RA leads to tissue contraction due to cicatricial retraction and muscular contractures^25,27,29^, while an insufficient restoration of the leg length was reported after reimplantation^20,27,29,51^. Further parameters like COR or FO, which can also influence the geometry, have not been analyzed until today.

Although a significant change of the hip geometry (especially LL and FO) was also observed in our study cohort after the RA period, in contrast to the above-mentioned studies our results indicate that a sufficient restoration of the final hip joint geometry (all measured radiographic parameters: FO, h/vCORD and LL) can be achieved after reimplantation. Considering the fact that PJI is a severe complication, which requires a time-consuming therapy with an extensive surgical repair, we observed only minor changes of the geometry after reimplantation in comparison to the initial pre-replacement status of the hip joint. The extensive leg length discrepancy (LLD) after RA (mean LLD: 54.1 mm) was significantly reduced after reimplantation (mean LLD: 8.3 mm), while this observation is consistent with the previously published results showing a LLD reduction from 46.4 to 7.9 mm after reimplantation^27^. Certainly, a LLD is frequently described as a source of patients` dissatisfaction^38,39^, but a LLD less than 10 mm is reported to be well tolerated by patients^40,52^.

We have also observed a significant change of the hip alignment after the RA period, with a mean FO increase of 3 mm (+ 6%). After reimplantation, the hip joint was realigned and a slight difference with a FO reduction of 2.7 mm (− 5.5%) was observed in comparison to the initial pre-replacement status of the hip joint. In general, studies reported that changes of the preexisting anatomical FO of about 15% can cause a limp, require the use of a walking aid and induce instability of the hip joint^34,35^. The observed changes of the FO in our study were considerably lower. In summary, a sufficient restoration of the LL as well as of the FO was reached after reimplantation.

Due to the fact that LL and FO changes can also result from a malposition of the femoral or/and acetabular component, the restoration of the horizontal and vertical acetabular center of rotation distance (h/vCORD) was evaluated. The position of the acetabular cup determines the COR of the hip joint. The consequences of implant malposition can include increased wear with higher component loosening^30,42–46^ and impaired muscle function^30–33^ resulting in poor functional outcome^36,37^. It has been reported that a cranialization of the COR of up to 13 mm and a medialization of 7.5 mm have no clinical consequence^53^. Other studies reported that a vCORD more than 20 mm does not affect the functional outcome, the range of motion or abductor muscles tension^33,54^, but is associated with an increased risk of aseptic loosening and should be avoided^55,56^. In our study, a COR cranialization within an acceptable range^57^ of 7.3 mm was registered in comparison to the initial vCORD of the pre-replacement hip joint. Furthermore, we could show a good restoration of hCORD (increase of 0.2 mm), while an optimal restoration of ± 5 mm is described in the literature^36,55,58^. The observed COR cranialization and lateralization is due to the acetabular reconstruction with individually selected implanted components in dependence of the occurring defect during PJI treatment.

In summary, the various radiographic parameters of the hip joint geometry were not influenced by two-stage exchange procedure with intermediate RA and an adequate restoration was observed after reimplantation.

Even though all measured radiographic parameters were within the normal range after reimplantation, deviations, especially regarding the LL and the vCORD, were registered compared to the initial pre-replacement parameters of the hip joint. After reimplantation we found a difference regarding the LL (mean reduction of 8.3 mm) and the vCORD (mean increase of 7.3 mm), but not regarding the FO or the hCORD. This difference may be due to the two-stage exchange procedure with intermediate RA, but in order to make an accurate statement about the impact of RA on the final hip geometry restoration after reimplantation, the knowledge of previous radiographic changes between preTHA and primary total hip arthroplasty (pTHA) is essential.

Interestingly, the above-mentioned differences of the LL and the vCORD after reTHA were already registered after pTHA with a mean LL reduction of 6.3 mm and a mean vCORD increase of 6.1 mm. This new aspect highlight that the radiographic parameters were not significantly influenced by the two-stage exchange procedure with intermediate RA while they have been previously changed after pTHA. The changes after pTHA are also reflected on the exemplary standardized radiographic images in Fig. 1.

Finally, the analysis of the radiographic parameters between the preexisting hip geometry after pTHA and the final reTHA showed an equivalent reconstruction with a negligible difference (LL: reduction of 2.0 mm, FO: reduction of 1.5 mm, hCORD: increase of 1.8 mm, vCORD: increase of 1.2 mm). These novel and partially unexpected data suggest that RA temporarily alters the radiographic parameters, but finally, has no impact on the restoration of the hip geometry after reimplantation.

Furthermore, the restoration of the FO and the LL was also independent of RA duration. The mean RA duration of 10.9 months (range: 1.8–72.1) in our study is presumably long enough that muscle and tissue contraction can be developed. However, our observation is in line with the results by Garcia-Rey et al. who showed a reduction of the leg length discrepancy to 7.9 mm after reimplantation^27^, although they examined a longer RA duration (mean: 39 months, range: 12–216). The important aspect that the final geometry restoration is independent of RA duration clarifies that PJI eradication can be accomplished without time pressure, while the occurring tissue contraction has to be addressed and surgically released during reimplantation. In contrast, Sigmund et al. showed that an increase of the RA duration resulted in an increase of the leg length discrepancy after reimplantation (RA duration < 10 weeks: median LLD of 13 mm, range: 10–20 mm) and RA duration > 10 weeks: median LLD of 20 mm, range: 10–35 mm)^51^. A perfect restoration of the final hip geometry depends on a sufficient surgical periarticular tissue release. Furthermore, femoral as well as acetabular defects have to be addressed during reimplantation in combination with the use of specifically selected implants, which have to be adapted to the individual anatomical and biomechanical conditions of the patient. One possible explanation for the difference to our results could be an insufficient intraoperative surgical release. Furthermore, they did not evaluate the COR, which can also influence the LL or their study population had a higher grade of femoral or acetabular bone defects. However, the relation between bone defects and the restoration of the LLD in dependence of the RA duration should be investigated in future studies.

Lastly, we assume that the number of reoperations (n = 2.0, range: 0–8), which are required in some cases, undoubtedly governs tissue contraction during RA period. However, a significant impact on the final hip geometry could not be determined in our study population.

The present study has some limitations. Our study was only focused on the evaluation of radiographic parameters concerning the hip joint restoration. Therefore, we did not evaluate the final hip function after the two-stage exchange procedure. Incidentally, Schröder et al. concluded that the improvement in hip function following reimplantation was marginal and the results were comparable to a well-functioning RA^59^. A further limitation was that we did not evaluate the amount of soft tissue damage or differences in the restoration of the radiographic parameters regarding the acetabular or femoral defect, but independent of the defect type we observed an adequate restoration of the final hip geometry. The study indicates that RA had no significant impact on the final geometry restoration of the hip joint. However, a subsequent prospective study powered to determine the effect of various parameters such as time with RA and re-operations would be needed to confirm our observations. Time intervals would need to be more defined for the time during RA (e.g. early conversion from RA: < 3 months, mid-term conversion: 3–6 months, 6–12 and > 12 month, with likely larger sample size to populate each interval.

## Conclusion

Our data revealed a good restoration of the hip joint geometry after reimplantation, while two-stage exchange procedure with intermediate RA did not alter the preexisting radiographic hip parameters during PJI treatment and had independent of RA duration or the number of reoperations no impact on the final hip geometry restoration.

### Ethics approval

This study was approved by the local ethics committee (Ethik-Kommission der Universitätsmedizin Göttingen, approval no. 22/12/17) and was carried out according to existing rules and regulations of the University Medical Center Göttingen.

### Informed consent

Informed consent was obtained from all subjects.
